# Can Ultrasound-Guided Xenon Delivery Provide Neuroprotection in Traumatic Brain Injury?

**DOI:** 10.1089/neur.2021.0070

**Published:** 2022-02-22

**Authors:** Misun Hwang, Rajarshi Chattaraj, Anush Sridharan, Samuel S. Shin, Angela N. Viaene, Sophie Haddad, Dmitry Khrichenko, Chandra Sehgal, Daeyeon Lee, Todd J. Kilbaugh

**Affiliations:** ^1^ Department of Radiology, Children's Hospital of Philadelphia, Philadelphia, Pennsylvania, USA.; ^2^ Department of Radiology, Perelman School of Medicine, University of Pennsylvania, Philadelphia, Pennsylvania, USA.; ^3^ Department of Chemical and Biomolecular Engineering, University of Pennsylvania, Philadelphia, Pennsylvania, USA.; ^4^ Department of Neurology, Perelman School of Medicine, University of Pennsylvania, Philadelphia, Pennsylvania, USA.; ^5^ Department of Pathology, and Laboratory Medicine, Children's Hospital of Philadelphia, Philadelphia, Pennsylvania, USA.; ^6^ Department of Pathology and Laboratory Medicine, Perelman School of Medicine, University of Pennsylvania, Philadelphia, Pennsylvania, USA.; ^7^ Department of Anesthesiology and Critical Care Medicine, Children's Hospital of Philadelphia, Philadelphia, Pennsylvania, USA.

**Keywords:** microbubbles, neuroprotection, traumatic brain injury, ultrasound, xenon

## Abstract

Traumatic brain injury (TBI) is associated with high mortality and morbidity in children and adults. Unfortunately, there is no effective management for TBI in the acute setting. Rodent studies have shown that xenon, a well-known anesthetic gas, can be neuroprotective when administered post-TBI. Gas inhalation therapy, however, the approach typically used for administering xenon, is expensive, inconvenient, and fraught with systemic side effects. Therapeutic delivery to the brain is minimal, with much of the inhaled gas cleared by the lungs. To bridge major gaps in clinical care and enhance cerebral delivery of xenon, this study introduces a novel xenon delivery technique, utilizing microbubbles, in which a high impulse ultrasound signal is used for targeted cerebral release of xenon. Briefly, an ultrasound pulse is applied along the carotid artery at the level of the neck on intravenous injection of xenon microbubbles (XeMBs) resulting in release of xenon from microbubbles into the brain. This delivery technique employs a hand-held, portable ultrasound system that could be adopted in resource-limited environments. Using a high-fidelity porcine model, this study demonstrates the neuroprotective efficacy of xenon microbubbles in TBI for the first time.

## Introduction

Traumatic brain injury (TBI) is the leading cause of death and morbidity in children and young adults.^1,2^ The neurological sequelae of TBI are affected not only by the type and severity of injury (primary injury) but also by a complex secondary pathophysiological cascade (secondary injury).^3–8^ For instance, TBI-induced axonal shear stretch results in the opening of voltage-gated calcium channels, triggering mitochondrial dysfunction, bioenergetic failure, and cell death pathways.^9–14^

There is a critical need to advance effective therapies that can be instituted quickly to prevent the progression of brain injury immediately after injury. At present, the standard of care is supportive in nature, focused on managing symptoms rather than managing the disease. Surgical intervention is instituted only in the case of severe, life-threatening brain injuries.

Xenon is an anesthetic gas with demonstrated neuroprotective effect in small animal models of TBI.^15–17^ Neuroprotection by xenon has also been confirmed using a blast injury model of *in vitro* TBI with reduced injury as assessed by propidium iodide staining.^15^ In a mouse model of controlled cortical impact, inhaled xenon administration at 75%^16^ and 30-50% of the total gas composition^17^ were shown to diminish injury extent and improve neurological outcomes. Histologic analyses demonstrated reduced astrogliosis and microglia damage in the hypothalamus and amygdala, respectively. In addition, there was an attenuation of hippocampal neuronal loss.^15^ Further, while not in TBI, a randomized clinical trial demonstrated that inhaled xenon decreased cerebral white matter damage, measured by magnetic resonance imaging (MRI), in 110 comatose survivors of out-of-hospital cardiac arrest.^18^

One reported mechanism of xenon-mediated neuroprotection is the antagonism of N-methyl-d-aspartate receptor (NMDA) subtype of glutamate receptors.^18,19^ As a potential neuroprotectant, xenon is hemodynamically safe, permeable to the blood–brain barrier, and has a wide safety margin *in vivo.*^20–23^ Unfortunately, the extremely high cost and inconvenience of xenon inhalational delivery limits its widespread study and use clinically.^24^ Likely, the xenon inhalation delivery method achieves low xenon concentration in the brain, limiting its neuroprotective efficacy without high dose concentrations. In addition, systemic delivery of high doses of xenon increases the likelihood of unwanted side effects such as vomiting and nausea.^25,26^

It is important to recognize that, to our knowledge, the neuroprotective effect shown in the rodent TBI studies has never been shown in large animal TBI models or in humans. Often, the therapeutic efficacy of novel drugs as determined from small animal models is not translatable to large animals or humans because of the marked differences in body habitus and drug pharmacokinetics.

To this end, our group has been developing a novel delivery technique that employs ultrasound-mediated release of xenon from microbubbles (MBs) into the brain via a localized application of a high acoustic impulse to the common carotid arteries after intravenous injection of xenon MBs (XeMBs). The overarching goal is therefore to overcome current limitations in xenon delivery by devising a novel, clinically translatable ultrasound-guided delivery method that improves neurological outcomes after TBI in a high-fidelity large animal model of focal TBI.

## Methods

### Formulation and imaging of XeMBs

The XeMBs were formulated as described previously.^27^ Briefly, 10 mL of a 10 mg/mL solution of DBPC (dibehenoylphosphatidylcholine) and DSPE-PEG5000 (in a 9:1 molar ratio) was purged with xenon gas for 10 min, followed by probe-sonication (Branson Digital Sonifier 250, 70% power) to create MBs. The MBs were then size separated by centrifuging at 300 *g* for 5 min. For making control MBs, perfluorobutane (PFB) was used instead of xenon, and a starting solution of 3 mg/mL lipids were used. The MBs were stored at 4°C in a glass vial with the headspace purged with xenon or PFB, as applicable. The MBs were used within 16–24 h of preparation.

### Neuroprotective efficacy of XeMBs in porcine model of TBI

The study was performed under an approved Institutional Animal Care and Use Committee protocol. A well-established focal controlled cortical impact porcine model of TBI^28–30^ (1-month old, weighing approximately 10 kg) was utilized to assess the therapeutic efficacy of PFB MBs versus XeMBs. In brief, the right coronal suture was exposed, and a craniectomy performed over the rostral gyrus allowing a 1 cm margin around the indenter tip of the cortical impact device described previously.^31^ The exposed dura was opened in a stellate fashion to reveal the cortical surface and the device stabilized against the skull with screws. The spring-loaded tip rapidly (4 msec) indented 0.63 cm of the cortical rostral gyrus.^31^

Day 1 and Day 5 MRIs were performed and conventional T1- and T2-weighted sequences analyzed to assess for changes in injury volume size in the two experimental groups (*n* = 6 total, 3 in each group). Day 5 MRI is obtained to validate the therapeutic efficacy of XeMBs into the subacute period. The experimental design consisted of intravenous PFB MB or XeMB administration at 1, 3, and 24 h after injury using the following protocol per treatment session: continuous infusion at 0.2 mL/min for 1.5 min, 0.4 mL/min for 0.5 min, and 0.6 mL/min for 6 min, for a total of 8 min of infusion. Injected MB concentrations for both xenon and PFB were adjusted to similar levels via necessary dilutions to a final number of approximately 10^8^ - 2x10^8^ per mL. Note that the dosing has been adjusted for pigs based on a previously performed pre-clinical study using rodents.^32^

A hand-held portable ultrasound device and probe (Lumify, Philips Healthcare) with a mechanical index of 1.0 was used to release the xenon gas from MBs at the level of the carotid artery, in the longitudinal axis at a frame rate between 18–20 frames per second and with a frequency range of 5–10 MHz.

### MRI imaging

Day 1 and Day 5 MRIs were obtained on a Siemens 3T TRIO MRI research magnet (Siemens, Munich, Germany) using a standard knee coil. Anatomical images (T1-weighted, T2-weighted, and diffusion-weighted) were acquired. T1- and T2-weighted sequences were loaded into pMRI (www.parametricmri.com) software.^33^ After manual delineation of edema (T2 hyperintense) and core (T2 hypointense) borders on each slice, a three-dimensional volume of injury was calculated and recorded using the volume module of pMRI. All tracings were performed by the consensus of two blinded reviewers, one of whom was a board-certified radiologist.

### Histopathology

Anesthetized animals were sacrificed, and the brain was removed whole. The brains were then cut into standardized 5 mm coronal sections and fixed in 10% neutral buffered formalin. After a fixation period of at least two weeks, whole brain slices were processed, paraffin embedded, cut into 5 μm sections, and stained with hematoxylin and eosin (H&E).

Histopathology studies were performed by an experienced board-certified neuropathologist. The reviewer was blinded. The H&E-stained histological sections of both hemispheres were examined to assess for the location, contents, and extent of the lesion. Perivascular inflammation was scored as follows: 0 = no to rare inflammatory cells; 1 = a few inflammatory cells; 2 = cuff of inflammatory cells 1 cell layer thick; 3 = cuff of inflammatory cells >1 cell layer thick. Vascular changes including endothelial proliferation were also examined and scored as follows: 0 = no vascular changes; 1 = reactive endothelial cells; 2 = endothelial proliferation (>1 cell layer thick). Perivascular inflammation and vascular changes were scored in the ipsilateral cortex surrounding the lesion and in the contralateral hemisphere.

### Statistical analysis

An unpaired Student *t* test was used to compare outcomes across groups. Statistical significance was defined as *p* < 0.05.

## Results

We have previously formulated MBs encapsulating pure xenon gas and tested its stability and echogenicity.^34^ We found that long-chain phospholipids like DBPC, which is known to form a rigid, coherent, gel phase below its melting temperature (74°C)^35^ are especially suited to produce stable MBs.^36–38^ These MBs were storage-stable and echogenic under clinical ultrasound in tissue-mimicking phantoms, making them the first echogenic pure noble gas MB theranostic agent.^27,34^ The amount of xenon in the MB suspension, estimated via gas chromatography-mass spectrometry, is 4.5 μmol/mL (∼10^8^ XeMBs/mL).^34^

**FIG. 1.**
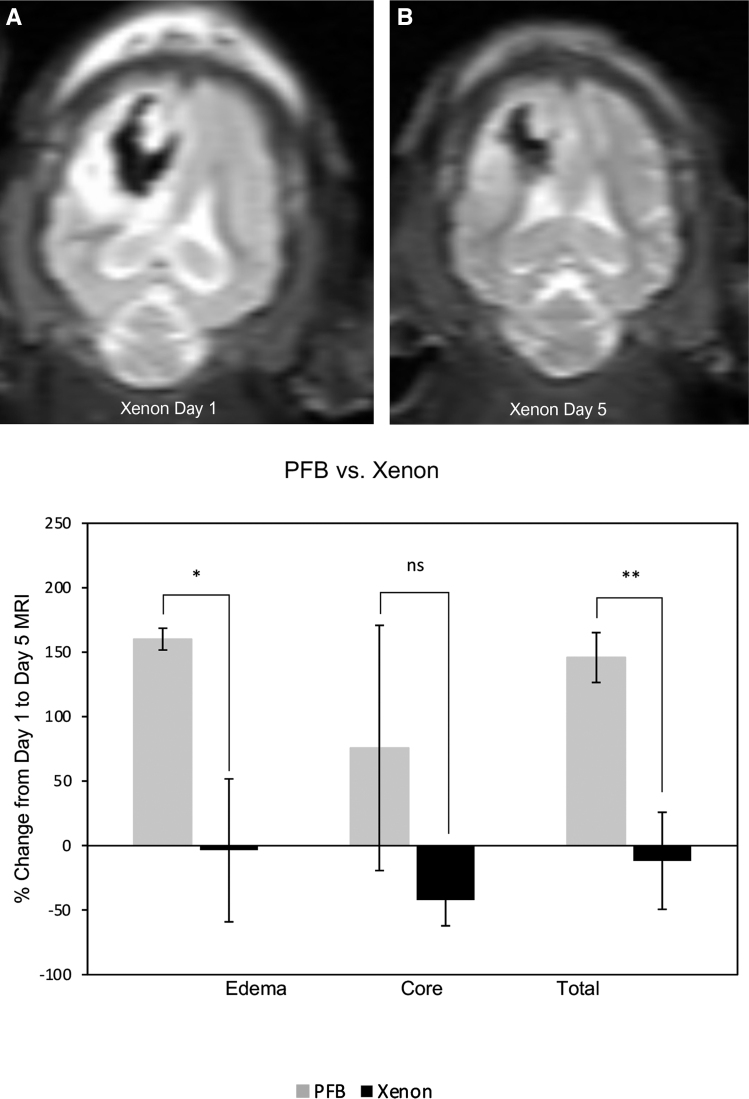
Neuroprotective efficacy of xenon microbubbles in the porcine model of traumatic brain injury. There is marked decrease in edema from Day 1 magnetic resonance imaging (MRI) (**A**) to Day 5 MRI (**B**) in the xenon-treated group. On the right, graph is showing percentage change in volumes (cm^3^) from Day 1 MRI to Day 5 MRI of edema, core, and total volume in the perfluorobutane (PFB)-treated (gray) and xenon-treated groups (*n* = 3 each). The statistical significance of the difference in the percentage change between PFB-treated versus xenon-treated groups is assigned as * for *p* ≤ 0.03, ** for *p* ≤ 0.01, and ns (not significant).

**FIG. 2.**
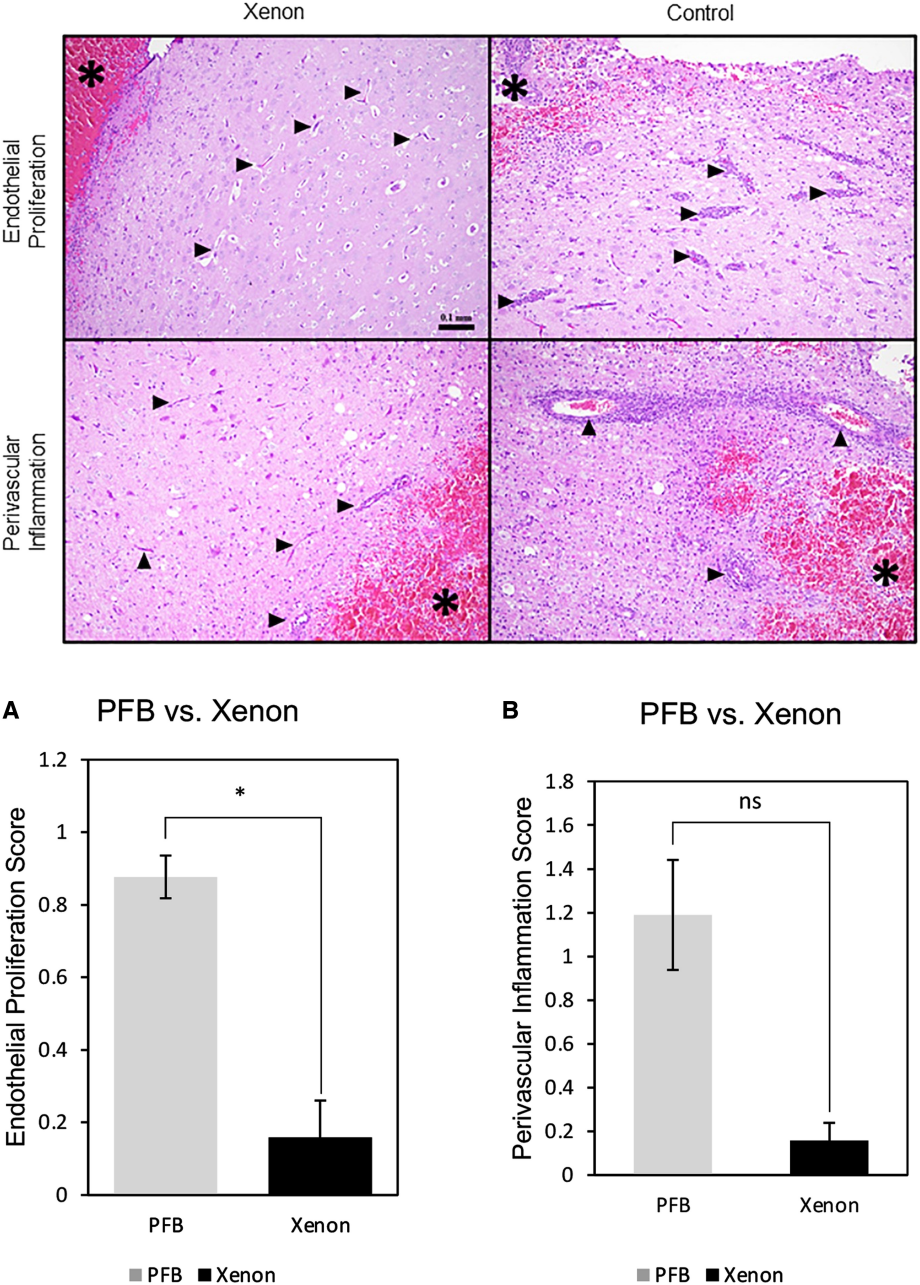
Histology in xenon microbubbles treated group versus perfluorobutane (PFB) microbubble treated group. Vessels (black arrows) adjacent to the site of injury (asterisks) in the xenon condition showed less endothelial proliferation and perivascular inflammation (including neutrophils, lymphocytes, and macrophages) compared with the PFB-treated group. Hematoxylin and eosin stain. Scale bar applies to all images. On the right, graphs are showing the difference in endothelial proliferation score (**A**) and perivascular inflammation score (**B**) between the PFB-treated (*n* = 2) and xenon-treated groups (*n* = 3). The statistical significance of the difference in scores between control vsversusxenon-treated groups is assigned as * for *p* ≤ 0.02 and ns (not significant).

Neuroprotective efficacy of XeMBs in the porcine model of TBI was confirmed on brain MRI (Fig. 1) and neuropathology (Fig. 2). On MRI, significant increase in injury edema (T2 hyperintense) (*p* = 0.03) and total volume (cm^3^) (*p* = 0.01) in the control group from Day 1 to Day 5 MRI was demonstrated, with increased perilesional high signal on T2-weighted sequence in comparison with the markedly decreased perilesional edema in the xenon-treated group. Moreover, an increase in size of the core/hemorrhage (T2 hypointense) was noted in the control group. This finding was not statistically significant, however.

Histological evaluation of the porcine brains demonstrated statistically significant decreased reactive vascular changes (*p* = 0.002) in the xenon-treated group compared with the control group. In addition, perivascular inflammation (including neutrophils, lymphocytes, and macrophages) adjacent to the site of injury was lower in animals treated with XeMBs in comparison with untreated, control animals. This was not a statistically significant finding, however.

## Discussion

This report proposes a new clinically translatable, cost-effective, and convenient therapy that can be used in the acute TBI setting. Ultrasound-guided xenon delivery to the brain in the acute post-TBI period drastically reduced injury edema and volume on MRI as well as perilesional reactive endothelial changes and perivascular inflammation on histology. Acoustically triggered release of xenon from its carrier MB at the level of the carotid artery using a portable, hand-held ultrasound system enables targeted cerebral delivery of xenon. Our finding provides a paradigm shift from the current standard of care, wherein no effective therapy exists for management of TBI in the acute setting.

While xenon gas inhalation therapy has been tried in animal models and humans, the approach typically used for administering xenon is expensive, inconvenient, and fraught with systemic side effects. Therapeutic delivery to the brain is significantly low, with much of the gas cleared via the lungs. Commercially available MBs used as ultrasound contrast agents for diagnostic imaging have an inert gas in the core (e.g., perfluorocarbons like PFB or perfluoropropane, sulfur hexafluoride, etc.), encapsulated by a phospholipid monolayer. Our XeMBs are not significantly different from commercially available MBs in terms of shell constituents and size, and thus have high clinical translatability.^39^ The ultrasound settings used are within the safety limitations of clinical use.

A recent publication has demonstrated that targeted release of xenon from xenon encapsulated liposomes can achieve neuroprotection,^32^ but data on large animal models, particularly a TBI model, are lacking. To this end, this study provides the first evidence toward the promising translational utility of XeMBs for neuroprotection in TBI.

The mechanisms of xenon-mediated neuroprotection in TBI are largely unknown, and our *in vivo* data will instigate exciting mechanistic studies for development of new therapeutic targets and drugs. Indeed, the multitude of xenon functions has been described in the literature. Our initial histological results also suggest interesting mechanisms with which xenon affects endothelial cell proliferation and perivascular inflammation in the acute post-TBI period.

Potential mechanisms of xenon-mediated neuroprotection include NMDA receptor subtype of glutamate receptor antagonism,^18,19^ potent activator of the two-pore domain K^+^ channel, shown to play an important role in neuroprotection,^40^ upregulation of the prosurvival proteins Bcl-2 and brain-derived neurotrophic factor,^41^ induction of the expression of hypoxia inducible factor 1α and its downstream effectors erythropoietin and vascular endothelial growth factor, and induction of trophic factors including brain derived neurotrophic factor.^41^

A larger randomized pre-clinical trial is warranted to validate the pilot findings presented. Moreover, determination of the neuroprotective efficacy of XeMB necessitates quantification of xenon concentration in blood and brain in the future. Future studies can help delineate XeMB pharmacokinetics, therapeutic dosing regimen, and biosafety to advance the novel clinical utility of XeMB for neuroprotection in TBI.

## Conclusion

Our data suggest that XeMBs can drastically reduce the injury extent as assessed using MRI and histology. This therapy could transform the way in which acute TBI is managed and can be broadly adopted. The data presented provide the first proof-of-concept evidence toward the use of XeMBs for management of acute TBI.

## Abbreviations Used

DBPC: dibehenoylphosphatidylcholine
H&E: hematoxylin and eosin
MB: microbubbles
MRI: magnetic resonance imaging
NMDA: N-methyl-d-aspartate
PFB: perfluorobutane
TBI: traumatic brain injury
XeMB: xenon microbubble
